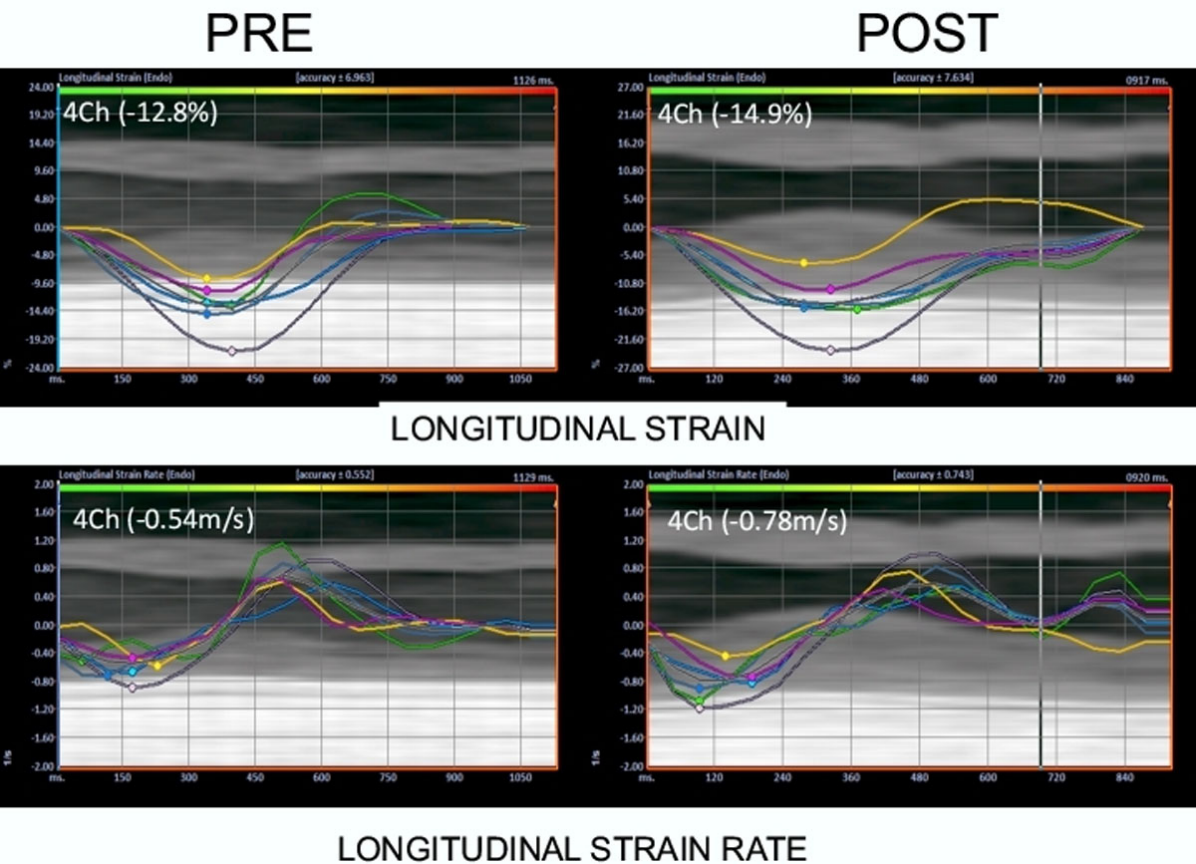# Failure to successfully open a chronic total coronary occlusion is associated with preserved global, but impaired regional myocardial function

**DOI:** 10.1186/1532-429X-15-S1-P224

**Published:** 2013-01-30

**Authors:** Lynne K Williams, Gideon A Paul, Idan Roifman, Mohammad I Zia, Bradley H Strauss, Andrew M Crean, Alexander W Leber, Alexander J Dick, Graham Wright, Kim A Connelly

**Affiliations:** 1 Medicine/Cardiology, St Michael's Hospital, Toronto, ON, Canada; 2 Medicine, Keenan Research Centre at the Li Ka Shing Knowledge Translation Institute, St Michael's Hospital, Toronto, ON, Canada; 3 Medical Biophysics, University of Toronto, Toronto, ON, Canada; 4 Schulich Heart Centre, University of Toronto, Toronto, ON, Canada; 5 Department of Medical Imaging, Toronto Congenital Cardiac Center for Adults, Toronto, General Hospital, University of Toronto, Toronto, ON, Canada; 6 Cardiology, Ottawa Heart Centre, Ottawa, ON, Canada

## Background

Non-randomized studies have reported a prognostic advantage with percutaneous coronary intervention (PCI) in the treatment of chronic total occlusions (CTO). However failure to cross and successfully open a CTO confers a worse clinical outcome. Quantitative assessment of global left ventricular (LV) function in those with failure to open a CTO has not demonstrated deleterious alterations in cardiac function/remodeling. We hypothesized that more sensitive techniques that assess regional wall motion, such as strain imaging, may identify segments where cardiac function is adversely affected in those with failure to open the CTO.

## Methods

30 patients referred for PCI to a single vessel de novo CTO underwent CMR examination before/after PCI. Left ventricular (LV) function and transmural extent of infarction (TEI) were assessed using standard cine SSFP and late gadolinium enhanced T1-weighted imaging on a 1.5T MRI system. Regional cardiac function was assessed from cine SSFP images using Velocity Vector Imaging software (VVI, Siemens) on 20 patients. Data from each segment was summated and presented as global longitudinal, circumferential and radial strain/strain rate. All data was analyzed blind to the outcome of PCI. Data is presented as mean +/- SD or percent change [delta (Δ)] in final value from baseline.

## Results

Successful CTO opening (TIMI 3 flow) occurred in 63% of patients. Ejection fraction (EF) increased post PCI within the successful group (Δ= +12 ± 20%), p < 0.0003). Within the failed group there was no change EF (p = 0.09). In those with dysfunctional but viable segments (25%) successful PCI increased EF (p = 0.04) in contrast to failed revascularization where the EF was not different.

Regional cardiac function varied significantly. Global longitudinal strain (GLS) fell significantly in the failed group (Δ=-25 ± 17%, p=0.02) in contrast to successful PCI where GLS did not change (Δ=+20 ± 32%, p=0.17). Global longitudinal strain rate followed a similar pattern to GLS (failed, Δ-30 ± 17% p<0.01 versus success Δ+25 ± 48% p=0.34). In contrast, radial and circumferential strain/strain rate were not different between groups after success/failed PCI (see table).

**Table 1:** Global and Regional function in CTO cohort

	Successful (S) CTO re-opening			Failed (F) CTO re-opening			S vs F
	Pre	Post	P	Pre	Post	P	P

Ejection Fraction (%)	50.3±12.6	54.3±10.7	0.0003	57.6±8.1	56.6±8	0.093	0.0001

Infarct size (%)	14.5±13.5	15.9±11.9	0.39	11.1±8.1	14.6±8.1	0.09	0.74

Global longitudinal strain (%)	-11 ± 4	-12 ± 3	0.17	-16 ± 3	-11 ± 4	0.02	0.02

Global longitudinal strain rate (m/s)	-0.63± 0.3	-0.69±0.2	0.34	-0.99 ±0.3	-0.70 ±0.3	0.01	0.01

Global circumferential strain (%)	-25 ± 8	-26 ±6	0.21	-29 ± 3	-29 ± 4	0.3	0.51

Radial strain (%)	23 ±8	24 ± 8	0.4	28 ±8	27 ± 3	0.7	0.31

Data is mean +/- SD.

## Conclusions

In this cohort, regional cardiac function assessment using Velocity vector imaging demonstrated a significant decline in GLS and GLSR in patients in whom PCI failed to open a CTO, with no change in global measures of cardiac function. Larger, randomized studies are required to assess the long-term benefits and morbidity of PCI in the treatment of CTOs, and potential impact of failure upon function and outcomes.

## Funding

Funding was provided in part by the CIHR, Canada.

**Figure 1:**